# Neuromonitoreo no invasivo en unidades de cuidados intensivos en Colombia

**DOI:** 10.7705/biomedica.5990

**Published:** 2021-12-15

**Authors:** Blair Ortiz, Sara Lanau

**Affiliations:** 1 Facultad de Medicina, Universidad de Antioquia, Medellín, Colombia Universidad de Antioquia Facultad de Medicina Universidad de Antioquia Medellín Colombia; 2 Sección de Neurología Infantil, Hospital San Vicente Fundación, Medellín, Colombia Sección de Neurología Infantil Hospital San Vicente Fundación Medellín Colombia; 3 Sección de Neurología Infantil, IPS Soytupediatra, Medellín, Colombia Sección de Neurología Infantil IPS Soytupediatra Medellín Colombia

**Keywords:** monitoreo, cuidado intensivo, electroencefalografía, análisis espectral, oximetría, Monitoring, critical care, electroencephalography, spectrum analysis, oximetry

## Abstract

El monitoreo electroencefalográfico no invasivo continuo es una técnica indispensable en los pacientes neurológicos críticos, ya que muestra de forma directa e indirecta su actividad cerebral y permite relacionar los hallazgos con su estado clínico. Es muy sensible, aunque su especificidad es menor, por lo que puede demostrar la alteración del estado de conciencia sin aclarar su etiología.

El uso del registro electroencefalográfico continuo en pacientes con alteraciones del estado de conciencia, convulsiones, o estado epiléptico convulsivo y no convulsivo, se ha incrementado en los últimos años porque permite obtener información en tiempo real de la función cerebral y de los cambios en el tiempo; además, facilita la detección de crisis epilépticas subclínicas y electrográficas, estas últimas de gran importancia, ya que no presentan correlación clínica.

Los hallazgos del monitoreo permiten modificar el tratamiento farmacológico y anticonvulsivo, y constituyen una gran ventaja para el médico tratante en la toma de decisiones oportunas que redunden en la mejoría del pronóstico del paciente.

## Abstract (en)

Continuous non-invasive electroencephalographic monitoring is an essential technique for critical care patients as it shows directly and indirectly the patient’s brain activity and makes it possible to relate it with findings in the clinical status. It is highly sensitive, although its specificity is lower, so they can show alterations of the state of consciousness without clarifying the etiology.

Continuous electroencephalographic recording in patients with altered levels of consciousness, seizures, and convulsive and non-convulsive status epilepticus has been increasing in recent years as real-time feedback of the cerebral function shows evolution changes and allows for the identification of electric and subclinical epileptic seizures that are highly important since they do not have clinical correlations.

These findings in electroencephalographic monitoring also help to modify pharmacological and antiseizure treatments. For practitioners, they are advantageous when making timely decisions that impact the prognosis of the patient.

Las diferentes modalidades de monitoreo neurológico no invasivo en las unidades de cuidados intensivos permiten que el médico no neurólogo tenga una aproximación diagnóstica muy certera, y pueda intervenir para evitar y tratar las complicaciones más frecuentes de los pacientes en estas unidades ^1^. Aunque son de gran utilidad para complementar el seguimiento neurológico, en muchas ocasiones se requiere de estudios más avanzados para caracterizar la enfermedad del paciente.

Estas técnicas difieren del monitoreo electroencefalográfico invasivo empleado en pacientes con epilepsia potencialmente remediable con cirugía. En esta actualización, se hará una revisión práctica de las modalidades de monitoreo neurológico no invasivo disponibles en Colombia, sin referencia al neuromonitoreo invasivo.

## Duración del neuromonitoreo

La duración ideal del monitoreo no invasivo debe ser de 24 horas como mínimo, pues los estudios muestran que el 50 % de las crisis se detectan en la primera hora del monitoreo y el 90 % durante las 24 horas iniciales. Si el tiempo de monitoreo es menor, las crisis epilépticas pueden no detectarse, lo que aumenta las complicaciones neurológicas y la morbimortalidad. Sin embargo, los expertos coinciden en que la duración del monitoreo electroencefalográfico debe individualizarse con base en el porcentaje de crisis según el tiempo de observación ^2^.

## Modalidades de neuromonitoreo no invasivo

Entre las diferentes modalidades de evaluación de la actividad cerebral, están el electroencefalograma de amplitud integrada (*ambulatory Electroencephalography*), el monitoreo de la función cerebral (*Cerebral Function Monitor*), el análisis del índice biespectral y la matriz de densidad espectral.

Una de las grandes ventajas de este tipo de monitoreo neurológico electroencefalográfico no invasivo, es la elevada correlación de sus medidas con las del electroencefalograma convencional, que sigue siendo la prueba de referencia para el diagnóstico en el paciente neurológico crítico. Esto quiere decir que las anormalidades observadas mediante estas técnicas deberían validarse con un electroencefalograma convencional (cuadro 1).

**Cuadro 1:** Indicaciones del monitoreo electroencefalográfico no invasivo en unidades de cuidados intensivos

Trastorno agudo de la conciencia (encefalopatía)
Lesión cerebral aguda
Estado epiléptico
Hemorragia subaracnoidea
Sospecha de crisis epilépticas
Bloqueo neuromuscular con lesión cerebral
Trauma craneoencefálico moderado a grave
Posterior a reanimación cardiopulmonar
Ahogamiento
Encefalopatía epiléptica (metabólica o genética)
Neurocirugía
Asfixia perinatal
Cirugía cardiovascular
Anestesia con soluciones para cardioplejia
Hipotermia local o corporal total
Síndrome de muerte súbita del lactante
Trauma no accidental (sospecha de maltrato infantil)

## Análisis del índice biespectral

En el paciente neurológico crítico, es fundamental conocer el grado óptimo de sedación y analgesia. En el coma anestésico empleado en los estados epilépticos, por ejemplo, debe conocerse el grado de compromiso del estado de conciencia y la titulación de los medicamentos sedantes.

El análisis del índice biespectral es un método cuantitativo que se basa en la actividad electroencefalográfica y determina la profundidad anestésica del paciente por medio de un valor numérico ^3^. Frecuentemente, se usa en el ámbito de la anestesia cardiovascular. Este índice permite añadir variables a partir de otros canales del electroencefalograma, como la matriz de densidad espectral que se describe más adelante.

Cuando la anestesia es guiada por el índice biespectral, se puede reducir el riesgo de despertares o estados de alerta durante la cirugía, en comparación con el monitoreo de los signos clínicos. En el paciente en estado epiléptico, permite monitorizar la continuidad del patrón de estallido-supresión y determinar más fácilmente la necesidad de hacer una rápida modificación del tratamiento ^4^. En el cuadro 2 puede observarse la cuantificación del estado de alerta y profundidad anestésica en el índice biespectral.

**Cuadro 2:** Representación cuantitativa del estado de alerta o de anestesia en el índice biespectral

Valores del IBE	Grado de hypnosis
100	Despierto
80-100	Responde a la voz
	Responde al hablar en voz alta o ante movimiento
60-80	Sedación moderada
40-60	Baja probabilidad de recuerdo explícito Falta de reacción a los estímulos verbales
	Sedación profunda
0-20	Estallido-supresión
0	EEG plano

IBE: índice biespectral; EEG: electroencefalograma

## Matriz de densidad espectral

**Figura 1:**
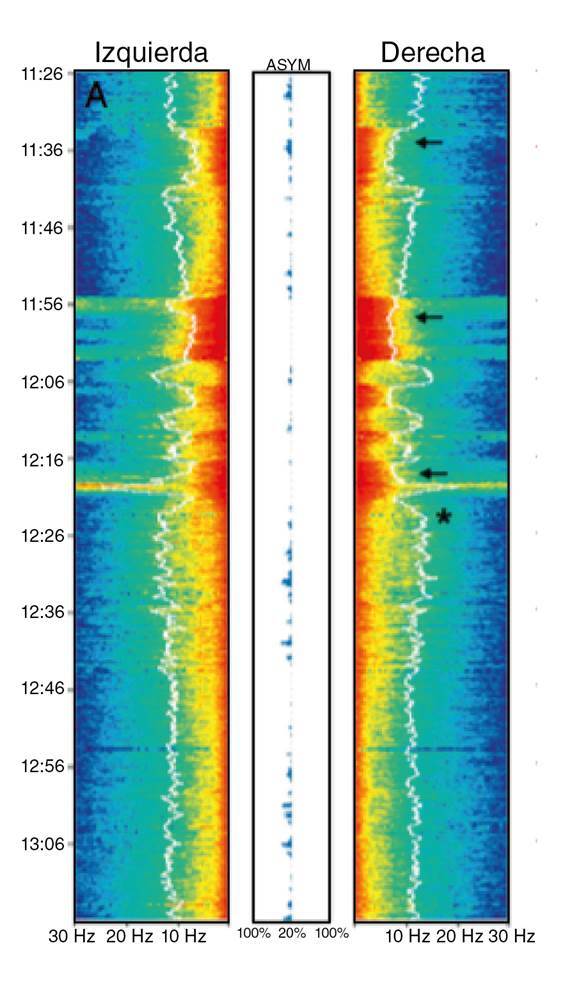
Análisis espectral comprimido (CSA) (Philips Intellivue Patient Monitor). Se muestra el análisis de un panel para el hemisferio izquierdo y otro para el derecho; el eje vertical organiza el tiempo, el horizontal, la frecuencia (en Hz) y, en la mitad, aparece el porcentaje de asimetría

La matriz de densidad espectral representa las frecuencias de las ondas cerebrales de forma continua en un gráfico de colores extraído del análisis electroencefalográfico, el cual se conoce como análisis espectral comprimido (*Compressed Spectral Array*). Este último muestra el rango de frecuencias predominantes desde el color azul (mínima frecuencia) hasta el rojo oscuro (máxima frecuencia) (figura 1).

La mayoría de los monitores que muestran la matriz de densidad espectral permiten observar el registro electroencefalográfico para que el neurólogo aprecie la actividad dominante, la lentificación y la asimetría de frecuencia interhemisférica ^5^.

**Figura 2:**
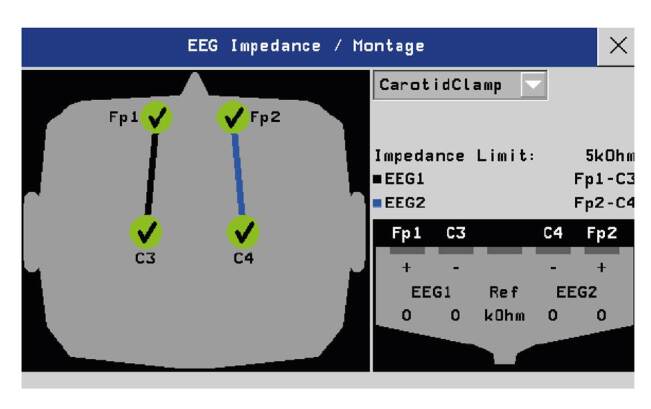
Monitor electroencefalográfico de dos canales de marca Nihon Kohden, modelo BSM- 2354A.

EEG1: canales izquierdos; EEG2: canales derechos; FP1: electrodo frontopolar izquierdo; FP2: electrodo frontopolar derecho; C3: electrodo central izquierdo; C4: electrodo central derecho; Ohm: (ohmio)

**Figura 3:**
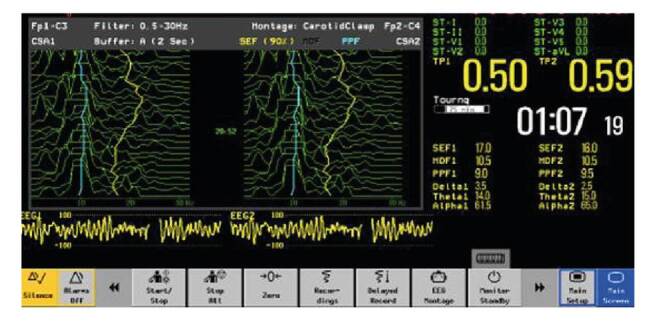
Monitor electroencefalográfico de dos canales de marca Nihon Kohden, modelo BSM- 2354A

EEG1: canales izquierdos; EEG2: canales derechos; SEF1: MDE izquierda; SEF2: MDE derecha; Delta/Theta/ Alpha: porcentaje de poder en cada banda; CSA: análisis espectral comprimido

En la figura 2 se muestra un montaje frontopolar-central a partir del cual puede visualizarse el electroencefalograma convencional de dos canales y la matriz de densidad espectral. Esta última hace referencia a la frecuencia encontrada en 90 a 95 % del muestreo del electroencefalograma. Por ejemplo, en la figura 3 se ve la de un paciente a quien se le practicó una sujeción de la arteria carótida derecha; la matriz de densidad espectral al 90 % es de 16 Hz en el lado derecho, lo que quiere decir que en el percentil 90 encontramos una frecuencia de 16 Hz en banda beta.

## Electroencefalograma de amplitud integrada

El electroencefalograma de amplitud integrada (aEEG) es una herramienta de monitoreo de pocos canales, usualmente dos, que permite visualizar el funcionamiento de los hemisferios cerebral derecho e izquierdo (montaje trasverso), o el polo anterior y el posterior (montaje longitudinal). Este tipo de electroencefalograma se usa principalmente en la etapa neonatal ^6^. Es de gran utilidad para determinar el enfoque diagnóstico inicial y el seguimiento del tratamiento en los pacientes con encefalopatía asfíctica y sospecha de actividad ictal; sin embargo, no reemplaza el electroencefalograma convencional y su sensibilidad para detectar crisis ictales es más baja ^7^. Tiene gran valor para orientar el pronóstico neurológico a largo plazo en pacientes con hipotermia terapéutica o sin ella.

**Figura 4:**
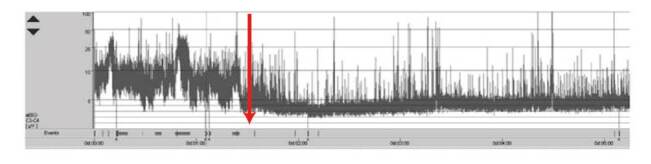
Monitoreo de la función cerebral de un neonato con ciclo de sueño y vigilia, y aumento de la discontinuidad e inicio de actividad ictal de bajo voltaje (a partir de la flecha). El eje vertical representa el voltaje en uV y, el horizontal, el transcurso del tiempo. Olympic Brainz Monitor v3.1.5

La interpretación se basa en la apreciación de las anormalidades paroxísticas y el trazado de fondo que sugieren la actividad ictal y el ritmo de la encefalopatía, respectivamente. En la figura 4 se muestra un monitoreo de la función cerebra de un recién nacido a término con encefalopatía neonatal y actividad ictal de inicio abrupto.

## Espectroscopia cerebral de infrarrojo cercano

La espectroscopia cerebral de infrarrojo cercano (*Near-Infrared Spectroscopy*) es una técnica de monitoreo no invasivo que mide la perfusión cerebral en tiempo real y detecta la saturación de oxígeno tisular con espectroscopia por transiluminación ^8^. Esta espectroscopia presenta un valor del porcentaje de saturación de oxígeno venoso en cada hemisferio cerebral; su valor normal está en el rango de 60 a 75 % y es francamente anormal por debajo de 55 %.

**Figura 5:**
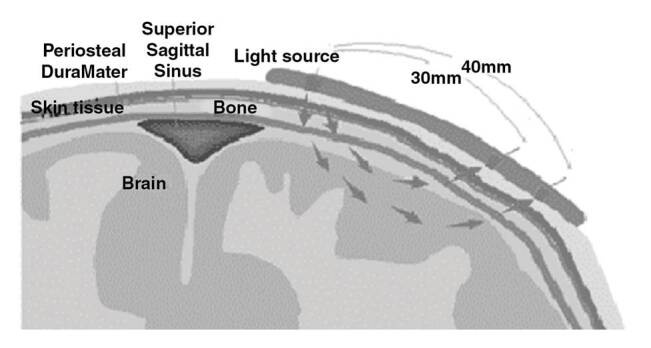
Fundamento fisiológico de la espectroscopia cerebral de infrarrojo cercano. Una fuente emite una luz que impacta los tejidos subyacentes y un detector mide el cambio de frecuencia de la misma luz reflejada. El propósito de esta espectroscopia es la medición de la saturación de sangre venosa en las venas puente que atraviesan el espacio subaracnoideo

Esta modalidad brinda información clínica importante en diversas situaciones, especialmente en aquellas en que las manifestaciones clínicas pueden ser muy imprecisas. La disminución de la oxigenación cerebral se define en la espectroscopia cerebral de infrarrojo cercano como la caída en la saturación de oxígeno mayor del 20 % con respecto a la línea de base, lo cual sugiere alteración neuronal por hipoxia. En la figura 5 puede observarse la distribución espacial de la luz de esta espectroscopia atravesando las diferentes capas hasta llegar al cerebro.

Entre las aplicaciones clínicas de la espectroscopia cerebral de infrarrojo cercano están la cirugía cardiovascular, la endarterectomía carotídea, la neurocirugía y el cuidado intensivo neonatal. Esta técnica tiene una correlación muy estrecha con la saturación de oxígeno de la vena cava superior. Es una herramienta que ayuda a detectar episodios de isquemia cerebral transitoria y estructurada ^9^.

## Ecografía Doppler transcraneal y del nervio óptico

Hay dos aplicaciones de la ultrasonografía en el monitoreo del paciente neurológico crítico: la medición del diámetro de la vaina del nervio óptico y la ecografía Doppler trancraneal. Con el neuromonitoreo guiado por ecografía, se puede: detectar estenosis u oclusión de las arterias intracraneales, monitorizar la evolución de los pacientes con vasoespasmo posterior a una hemorragia subaracnoidea, detectar la embolia cerebral, evaluar el sistema de arterias colaterales cerebrales, complementar la evaluación del paciente con muerte cerebral, calcular indirectamente la presión intracraneal y de perfusión cerebral, y detectar la hemorragia subaracnoidea y los aneurismas cerebrales ^10^.

Este monitoreo no es invasivo ni doloroso, no emite radiación y puede hacerse al lado del paciente crítico ^11^. Se han descrito muy pocos riesgos para el paciente y el principal son las reacciones alérgicas al gel conductor que debe utilizarse con el transductor ecográfico.

Específicamente, la medición del diámetro de la vaina del nervio óptico y el análisis Doppler de la resistencia vascular son muy precisos para determinar el aumento de la presión endocraneal en el paciente con lesión cerebral aguda ^12^ e hipertensión arterial con repercusión sistémica ^13^. La ecografía Doppler intracraneal siempre debe registrar la velocidad del flujo y los índices de pulsatilidad de las arterias de la base del cerebro ^14^^,^^15^.

## Conclusión

El monitoreo electroencefalográfico no invasivo es una herramienta indispensable en todas las unidades de cuidado intensivo porque permite al médico no neurólogo diagnosticar y tratar oportunamente las complicaciones del paciente neurológico crítico. El conocimiento de las diferentes modalidades de monitoreo nuerolólgico es indispensable para decidir si la enfermedad es del sistema nervioso central o si es sistémica y, así, determinar la intervención terapéutica más precisa. Los resultados de las mediciones electrográficas siempre deben validarse con el electroencefalograma convencional, ya que sigue siendo la prueba de referencia.
